# Omphalocele prevalence and co-occurring malformations: a nationwide register-based study of Danish live births in 1997–2021

**DOI:** 10.1007/s00383-024-05897-5

**Published:** 2024-11-22

**Authors:** Ulrik Lausten-Thomsen, Paula L. Hedley, Kristin M. Conway, Katrine M. Løfberg, Lars S. Johansen, Paul A. Romitti, Michael Christiansen

**Affiliations:** 1 Department of Neonatology, Copenhagen University Hospital Rigshospitalet, Blegdamsvej 9, 2100 Copenhagen, Denmark; 2 Department for Congenital Disorders, Statens Serum Institut, Copenhagen, Denmark; 3 Department of Epidemiology, College of Public Health, The University of Iowa, Iowa City, Iowa USA; 4 Department of Pediatric Surgery, University Hospital Rigshospitalet, Copenhagen, Denmark; 5 Department of Biomedical Sciences, University of Copenhagen, Copenhagen, Denmark

**Keywords:** Congenital abnormalities, Epidemiological monitoring, Neonatal surgery, Neonatal biobanking, Omphalocele

## Abstract

**Purpose:**

Omphalocele is a congenital abdominal wall defect associated with a high risk of morbidity and mortality, with co-occurring congenital malformations often being the most important prognostic factor. High rates of spontaneous and medical terminations have been reported among pregnancies with omphalocele and co-occurring malformations. Few studies have focused on co-occurring malformations, particularly non-gastrointestinal malformations among live births. This study examined birth prevalence of omphalocele and co-occurring major malformations among a 25-year Danish liveborn cohort.

**Methods:**

This nationwide retrospective prevalence study used data from the Danish National Patient Register and Danish Civil Registration System for infants who were delivered in Denmark during 1997–2021 and included in the Danish neonatal screening biobank. Diagnoses of omphalocele and co-occurring malformations were ascertained and prevalence estimated using Poisson regression.

**Results:**

Among 1,498,685 live births, 147 infants with omphalocele were identified, yielding a combined and stable prevalence (per 10,000 infants) of 0.98 (95% CI 0.83–1.15). Over one-half (53.7%) presented with one or more major malformations, and an additional 17.0% were diagnosed with a syndrome.

**Conclusions:**

Omphalocele birth prevalence in Denmark was stable over a recent 25-year period. The proportion of infants with co-occurring major malformations or diagnosed syndrome has important implications for long-term healthcare demands.

**Supplementary Information:**

The online version contains supplementary material available at 10.1007/s00383-024-05897-5.

## Introduction

Omphalocele, or exomphalos, is a relatively rare abdominal wall defect defined as a congenital malformation characterized by herniation of abdominal contents through the umbilical insertion [1]. This herniation is covered by a membrane, which is most often intact but may be ruptured [1]. The severity of the defect varies in size and the amount of herniated viscera. In general, omphaloceles are categorized as small, without herniated liver, or large when involving herniation of the liver [2].

Although various theories have been proposed, there is no clear consensus on the exact etiology of the dysgenesis of the abdominal wall that leads to omphalocele [3, 4]. During normal embryogenesis, the midgut herniates out of the abdominal cavity into the umbilical cord thereby accommodating rapid growth and elongation of the midgut and its associated mesentery. By the 10th–12th weeks of gestation, the intestines rotate and return to the abdominal cavity. However, with omphalocele, this retraction fails due to improper formation and closure of the ventral body wall, resulting in the persistent herniation of abdominal contents a sac protruding from the umbilical base [2–4].

Omphalocele is associated with high morbidity and mortality rates, that are influenced significantly by the condition’s frequent association with other major malformations, including structural malformations and chromosomal abnormalities [5–7]. Omphalocele presents with several recognized syndromes, the most prevalent being Beckwith–Wiedemann syndrome and trisomies 13 and 18, and to a lesser degree trisomy 21 [2, 8, 9]; structural malformations co-occurring with omphalocele frequently affect the cardiac, renal, limb, and central nervous systems [2]. These co-occurring malformations can contribute to the high morbidity and mortality rates reported for omphalocele, with the degree of severity of co-occurring malformations often the most crucial prognostic factor [2].

The birth prevalence (per 10,000 live births) of omphalocele is reported to range from 0.56 to 4.85 according to international registers [10, 11] with no significant long-term trends observed [10–13]. Omphalocele is usually diagnosed antenatally by ultrasound [14, 15], and due to the co-occurrence of other major malformations or chromosomal abnormalities, there are significant rates of naturally occurring fetal demise as well as important rates of medical terminations, particularly in the western world [15–17]. In Europe, studies, mostly performed in single centers, have reported termination rates to exceed one-half of all individuals with diagnosed omphaloceles [15–18].

Birth prevalence estimates for omphalocele vary by region and center and depend on whether the data include all affected pregnancies (i.e. livebirths, still births, and terminations of pregnancies) or livebirths only. From a public health perspective, current data on the birth prevalence of omphalocele and co-occurring malformations in live births and early survivors are crucial, as these data most adequately represent the direct and ongoing burden on healthcare facilities and depict the most common long-term phenotypical presentation of individuals diagnosed with omphalocele. Also, understanding the types and proportions of co-occurring malformations in these individuals is essential for identifying the long-term care needs and resource allocation required for these patients. By focusing on a liveborn cohort, we can more accurately assess the long-term healthcare demands, including surgeries, therapies, and supportive care required throughout their lives.

Accordingly, the aim of our study is to estimate the birth prevalence of Danish infants with omphalocele born during 1997–2021 using publicly available data from nationwide registers. In addition, we estimated the prevalence of co-occurring major congenital malformations and chromosomal abnormalities and examined trends in annual prevalence over this 25-year period.

## Methods

### Study sample

In this nationwide retrospective study of the prevalence of omphalocele, the data were sourced from the Danish Biobank Register, which integrates information from the Danish Civil Registration System (including date of birth, social security number, and country of birth for individuals and their parents) with data from the Danish National Patient Register (including diagnostic codes, dates of diagnosis, and surgical intervention codes). This register encompasses all individuals with specimens stored in the Danish National Biobank since 1982.

Using the Danish Biobank Register’s online interface [19], entries for all infants born in Denmark during 1st January 1997 through 31st December 2021 who were sampled for neonatal screening were examined to identify those diagnosed with omphalocele (ICD-10-DK: DQ792—“Omfalocele”) within 1 year of birth. The coverage for neonatal screening in Denmark is nearly 100% [19], so this study cohort represents a nationwide cohort of all infants who survived to time of screening (days 5–7 in 1997–2008 and days 2–3 in 2009–2021).

### Case classification

Eligible infants with omphalocele were assessed for any malformations across all organ systems (ICD-10-DK: DQ00–DQ899 or any genetic syndrome (ICD-10-DK: DQ90–DQ999). Infants were classified as isolated (no additional, major congenital malformations) or as presenting with co-occurrence of one or more major malformations. Distinction between minor and major malformations was determined though discussion within the authors’ group and using classifications suggested by EUROCAT [11].

Gastroschisis is another congenital abdominal wall defect that may be mistaken for an omphalocele, particularly if the membrane that normally covers the omphalocele ruptures [20], as has been reported to occur in up to 15% of infants with omphalocele [21]. Therefore, to eliminate infants erroneously diagnosed as both having gastroschisis (ICD-10-DK: DQ793—“Gastoskise”) and omphalocele, those classified as having received surgery for gastroschisis (Danish SKS-code: DJAG10—“Operation for gastroskise”) were excluded. To further reduce possible diagnostic misclassification, the cohort was restricted to children born in Denmark, but with no limitations based on parental nativity.

Registry data obtained included information on infant sex (female or male) and parental nativity (both, one, or neither parent born in Denmark). Given its inherent contribution to significant morbidity and mortality, prematurity was also evaluated. Prematurity was classified based on the diagnosis of prematurity (ICD-10-DK: DP073—“Præmaturitet”), immaturity (ICD-10-DK: DP072—“Immaturitet”), extremely low birth weight (< 1000 g, ICD-10-DK: DP070—“Ekstremt lav fødselsvægt”), or very low birth weight (1000–1499 g, ICD-10-DK: DP071A—“Lav fødselsvægt”).

### Statistics

Statistical analyses were performed using R (version 4.3.0 “Already Tomorrow”). Birth prevalence of omphalocele and of co-occurring malformations were estimated by dividing the total number of infants with omphalocele by the number of live births who survived to neonatal screening (henceforth, screened infants), expressed per 10,000 screened infants. Prevalence rate ratios (PRRs) and 95% confidence intervals (CIs) were estimated using Poisson regression models. Chi-square with Yates’ continuity correction tests were employed to explore differences in omphalocele birth prevalence by sex, parental nativity, and prematurity. Temporal trends in prevalence were analyzed using Poisson regression models.

## Results

**Fig. 1:**
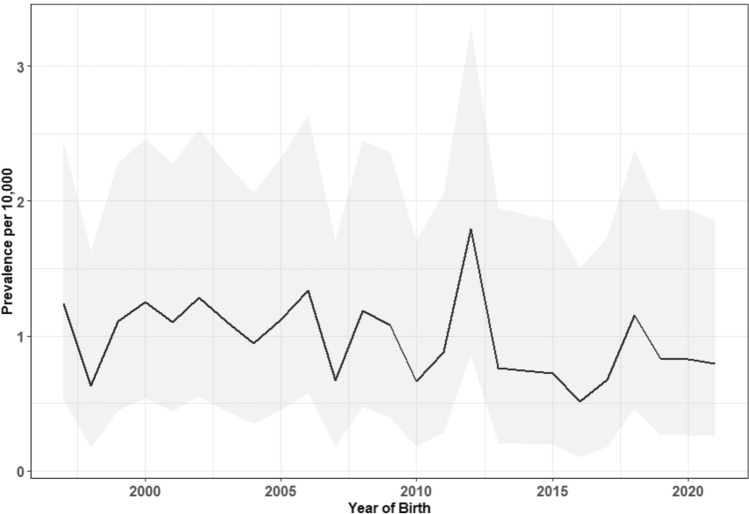
Omphaloceles in Denmark: omphalocele prevalence estimates (line) and 95%CIs (shaded area)

Of the 1,498,685 screened infants born in Denmark during 1997–2021, 768,964 (51.3%) were males and 729,721 (48.7%) were females. Among these infants, 147 were diagnosed with omphalocele, comprising 89 (60.5%) males and 58 (39.5%) females, for a male-to-female ratio of 1.5:1. The combined estimated birth prevalence (per 10,000 screened infants) of omphalocele was 0.98 (95% CI 0.83–1.15), with estimates of 1.16 (95% CI 0.93–1.42) and 0.79 (95% CI 0.60–1.03) for males and females, respectively (Fig. 1). A Chi-squared test indicated a statistically significant higher birth prevalence in boys (*χ*^2^ = 4.66, *p* = 0.031).

Annual observed rates of omphalocele and their 95% CI are presented in Fig. 1. Temporal trend analysis using Poisson regression showed a small, non-significant annual decline in overall omphalocele rates of 1.49% (*p* = 0.188) during the study period.

A subanalysis, comparing the overall birth prevalence estimates for the time-period from 2003 to forward, following the introduction of systematic prenatal ultrasound nationwide in Denmark, with that from the 1997 to 2002 time period produced no statistically significant difference in overall prevalence estimates (*X*^2^ = 0.637, *p* value = 0.425). Similarly, comparison of estimates for 2009–2021, when an infant completed neonatal screening on days 2–3, with that for 1997–2008, when the screening was completed within 5–7 days, again produced no significant difference (*X*^2^ = 1.401, *p* value = 0.237) (data not shown).

Although prematurity was present in 19.7% of infants with omphalocele (95% CI 13.2–28.3), significantly higher than the general population (*χ*^2^ = 56.814, *p* < 0.00001), no sex differences in prematurity rates were observed (*χ*^2^ = 1.681, *p* = 0.195).

Prevalence estimates for syndromes and co-occurring malformations are presented in Table 1. Overall, 25 (17.0%) infants with omphalocele were classified as syndromic. Of these 9 (6.1%) had chromosomal abnormalities, including autosomal trisomies (Down, Edward’s, or Patau’s syndrome), and 16 (10.9%) had a diagnosed syndrome, most commonly Beckwith–Wiedemann syndrome. Of the remaining 83.0%, 43 (29.3%) were classified as isolated and 75 (53.7%) were classified with one or more co-occurring malformations. The most frequent co-occurring malformations were cardiac, present in 34.0% of infants with omphalocele. Atrial septal defects and ventricular septal defects were the most common cardiac malformations. Other severe structural cardiac malformations diagnosed included tetralogy of Fallot, double outlet right ventricle, coarctation of aorta, and congenital pulmonary valve stenosis, all equally reported.

**Table 1:** Number and prevalence per 10,000 infants with omphalocele associated with syndromic and non-syndromic gastroschisis cases diagnosed in Denmark during 1994–2021

Phenotype	*N*	Prevalence per 10.000 (95% CI)	% Of primary omphalocele*
Omphaloceles (all types)	147	0.98 (0.83–1.15)	100
Syndromic	25	0.17 (0.11–0.25)	17.0 (11.0–25.1)
Chromosomal abnormalities	9	0.06 (0.03–0.11)	6.1 (2.8–11.6)
Other syndromes	16	0.11 (0.06–0.17)	10.9 (6.2–17.7)
Nonsyndromic	122	0.81 (0.68–0.97)	83.0 (68.9–99.9)
Isolated	43	0.29 (0.21–0.39)	29.3 (21.2–39.4)
Major co-occurring malformation	79	0.53 (0.42–0.66)	53.7 (42.5–67.0)
CNS (DQ00–DQ079)	5	0.03 (0.01–0.08)	3.4 (1.1–7.9)
EYES, EARS, FACE, AND NECK (DQ10–DQ189)	< 5	NE	NE
CARDIAO-VASCULAR (DQ21–DQ289)	50	0.33 (0.25–0.44)	34.0 (25.2–44.8)
RESPIRATORY (DQ30–DQ349)	< 5	NE	NE
ORO-FACIAL (DQ35–DQ379)	< 5	NE	NE
GASTROINTESTINAL (DQ38–DQ459)	33	0.22 (0.15–0.31)	22.4 (15.4–31.5)
URO-RENAL–GENITAL (DQ50–DQ649)	6	0.04 (0.02–0.09)	4.1 (1.5–8.9)
BONES, MUSCLES, AND TISSUES (DQ65–DQ799)	17	0.11 (0.07–0.19)	11.6 (6.7–18.5)
OTHER MALFORMATIONS (DQ80–DQ899)	31	0.21 (0.14–0.29)	21.1 (14.3–29.9)

Major co-occurring malformations are grouped as per ICD-10

*NE* not estimated

*A patient can have several co-occurring malformations and consequently the categories can sum up to more than 100%

When exploring the temporal changes in prevalence of major co-occurring malformations in infants with omphalocele, a decrease, although statistically non-significant, was seen in the last 5 years (*χ*^2^ = 2.792, *p* < 0.095). This tendency was more pronounced, although still not significant, when observing exclusively the most severe of the major co-occurring malformations (supplemental Fig. 1).

## Discussion

For omphalocele, terminations and early neonatal demise influence overall livebirth rates and associated malformation rates, and accordingly focus on the liveborn cohort is essential to assess long-term healthcare demands. In this nationwide, quarter-century cohort, we observed an overall stable omphalocele birth prevalence (per 10,000 screened infants) of 0.98, with a higher birth prevalence among males than females (male:female ratio of 1.5:1). These findings are similar to published estimates from Nordic countries [17, 22] and Europe [10]. The observed increased birth prevalence for males is equally well established, and although a recent meta-analysis [23] estimated the male:female ratio at 1.16, the 95%CI for our data encompasses this estimate. Any differences between our prevalence estimates and international estimates may reflect true regional and/or temporal differences; however, they may also reflect methodological differences, as our data exclusively included liveborn children included in the Danish Biobank Register.

Contrary to prevalence trends observed for gastroschisis [24], omphalocele birth prevalence remained unchanged over our study period. Any potential changes in liveborn omphalocele prevalence are at least partly influenced by potential changes in rates of elective terminations of pregnancy, which makes direct comparison to results from other regions complicated as other countries may not have the same juridical or cultural view on elective terminations. However, our finding of a relatively stable birth prevalence estimate is comparable to that for liveborn infants from a recent report from Sweden, that has a similar cultural attitude towards abortion [22]. Furthermore, we did not find a change in omphalocele birth prevalence when comparing the time period before and after the introduction of systematic prenatal ultrasound in Denmark, 2003, further supporting the overall stability of our omphalocele birth prevalence estimate.

We observed syndromes and/or chromosomal abnormalities in 17% of infants with omphalocele with Beckwith–Wiedemann syndrome being the most frequent diagnosis. Isolated chromosomal abnormalities were observed in 6.1% which is in concordance with, although slightly higher than, a comparable Scandinavian report [22]. The high proportion of infants with chromosomal abnormalities, syndromes, or major malformations was evident, with less than one-third having isolated omphalocele. This finding concurs with reports from other Nordic countries [17, 22] assumed to have similar prenatal screening and perhaps more importantly, similar juridical and cultural perception towards abortion. The latter is important as termination of pregnancies with omphalocele is quite common in Europe [15, 17, 22, 25, 26] particularly with co-occurring chromosomal abnormalities and other major malformations, as these abnormalities and malformations are believed to impact decisions to opt for pregnancy termination [15]. The observed decrease in co-occurring malformations, particularly in the most severe form of co-occurring malformations, in the most recent years of our study period may reflect better prenatal diagnostic and subsequent increased probability of elective terminations of pregnancies with omphalocele and major co-occurring malformation, such as chromosomal abnormalities.

A fundamental constraint of our study is that it exclusively encompasses infants that were alive during the neonatal screening sample collection. Consequently, with our study–design, we are unable to provide data on the prevalence of omphalocele in pregnancies that were terminated or resulted in fetal or very early neonatal demise. Yet, the infants with omphalocele included in this study are representative of those who engage with the Danish healthcare system, making it crucial to describe and understand this group. Our study is also limited by small number statistics; as omphalocele luckily remains a rare condition, small but true trends in birth prevalence may be impervious to statistical detection due to the larger variability in the estimates caused by the small number of affected infants.

There are several strengths of this study. The use of the Danish National Patient Register leverages a national, register-based methodology that captures comprehensive real-world clinical data thereby minimizing ascertainment bias. These data have nationwide coverage, with specific focus on individuals who have a dried bloodspot specimen stored at the DNSB, this means that we have access to diagnostic data for virtually all infants born in Denmark and thereby facilitates a comprehensive national evaluation of the epidemiological characteristics of this disease within Denmark. The prospective nature of the register data collection may offer advantages over the retrospective approach commonly used in many other studies. While these register data have not undergone formal validation, as might be seen in specialized malformations registries like EUROCAT and accordingly there is a possibility that a few infants with omphalocele could have been inaccurately reported, the Danish Health Registers are widely regarded as exceptionally reliable, consistently covering all demographics without disparities in gender, social group, or geography [27]. Moreover, the overall sensitivity of omphalocele registration in these registers is estimated to be very high, at 99% [28], making it highly unlikely that our findings are affected by any failure in ascertainment and reporting. In addition, given that the incidence of major co-occurring malformations in our study aligns with previous reports, it is improbable that our data suffer from any systematic underreporting.

In conclusion, we observed that the overall birth prevalence of omphalocele in Denmark has remained stable over the last quarter of a century, albeit with a non-significant decline. As our data show, omphalocele remains a rare major abdominal malformation with a relatively high degree of co-occurring malformations, even in live-born children surviving to neonatal screening. Accordingly, continuous surveillance of birth prevalence and associated malformations remain warranted to understand the care needs and resource allocation required for these patients.

## Supplementary Information

Below is the link to the electronic supplementary material.

Supplementary file1 Supplemental Fig. 1. Prevalence of observed rates (dots) of severe, major congenital abnormalities in five equal time-frames (1994–2001, 2002–2006, 2007–2011, and 2012–2021) with 95% CI (light gray). (TIFF 1574 KB)

## Data Availability

Data is provided within the manuscript or supplementary information files.

## Declarations

### Previous communication

This is the first submission of the manuscript to the journal. This manuscript or parts of it is not being considered for publication by another journal, nor has it been published or posted elsewhere.
