# Supplementary figures and images for: Omphalocele prevalence and co-occurring malformations: a nationwide register-based study of Danish live births in 1997–2021
Source: Pediatr Surg Int. 2024 Nov 22;41(1):1. doi: 10.1007/s00383-024-05897-5 (PMC11584431; doi:10.1007/s00383-024-05897-5)

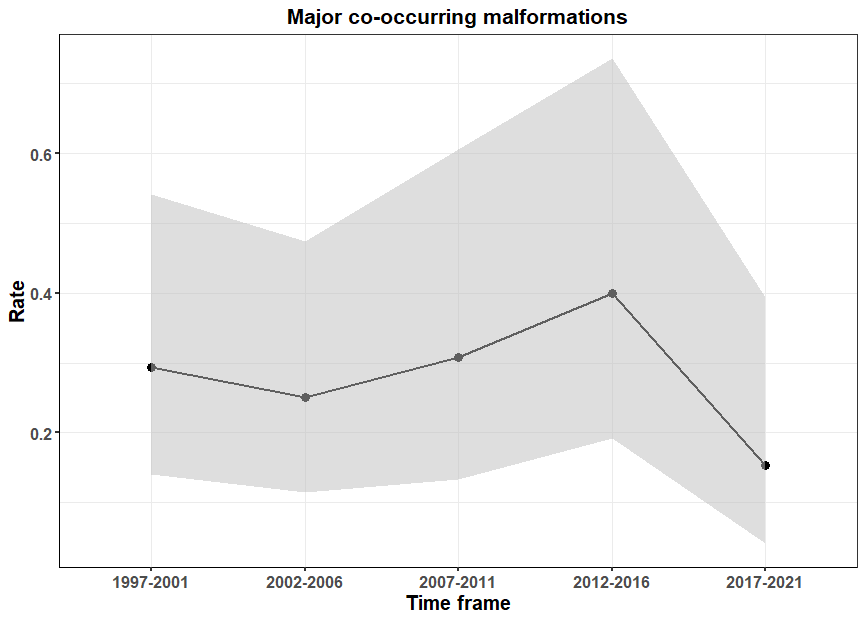

Supplement: Supplementary file 1 — Supplementary file1 Supplemental Fig. 1. Prevalence of observed rates (dots) of severe, major congenital abnormalities in five equal time-frames (1994–2001, 2002–2006, 2007–2011, and 2012–2021) with 95% CI (light gray). (TIFF 1574 KB) [file 383_2024_5897_MOESM1_ESM.tiff]
